# Speaker Recognition Using Constrained Convolutional Neural Networks in Emotional Speech

**DOI:** 10.3390/e24030414

**Published:** 2022-03-16

**Authors:** Nikola Simić, Siniša Suzić, Tijana Nosek, Mia Vujović, Zoran Perić, Milan Savić, Vlado Delić

**Affiliations:** 1 Faculty of Technical Sciences, University of Novi Sad, Trg Dositeja Obradovica 6, 21000 Novi Sad, Serbia; sinisa.suzic@uns.ac.rs (S.S.); tijana.nosek@uns.ac.rs (T.N.); miavujovic@uns.ac.rs (M.V.); vlado.delic@uns.ac.rs (V.D.); 2 Faculty of Electronic Engineering, University of Nis, Aleksandra Medvedeva 14, 18000 Nis, Serbia; zoran.peric@elfak.ni.ac.rs; 3 Faculty of Sciences and Mathematics, University of Pristina in Kosovska Mitrovica, Ive Lole Ribara 29, 38220 Kosovska Mitrovica, Serbia; milan.savic1@pr.ac.rs

**Keywords:** speaker recognition, convolutional neural network, quantization, emotional speech

## Abstract

Speaker recognition is an important classification task, which can be solved using several approaches. Although building a speaker recognition model on a closed set of speakers under neutral speaking conditions is a well-researched task and there are solutions that provide excellent performance, the classification accuracy of developed models significantly decreases when applying them to emotional speech or in the presence of interference. Furthermore, deep models may require a large number of parameters, so constrained solutions are desirable in order to implement them on edge devices in the Internet of Things systems for real-time detection. The aim of this paper is to propose a simple and constrained convolutional neural network for speaker recognition tasks and to examine its robustness for recognition in emotional speech conditions. We examine three quantization methods for developing a constrained network: floating-point eight format, ternary scalar quantization, and binary scalar quantization. The results are demonstrated on the recently recorded SEAC dataset.

## 1. Introduction

A typical speaker recognizer consists of two main components: a feature extraction module and a speaker modeling part. The feature extraction module transforms raw audio signals into feature vectors that capture speaker-specific properties. Based on these vectors, the speaker model is trained. Which features to extract and what technique to use for the modeling are strongly dependent on the intended application, computing resources, and amount of speech data available [1].

In the past several decades, many techniques for automatic speaker recognition (SR) have emerged—from the oldest non-parametric to statistical and state-of-the-art methods based on deep neural networks [2,3].

Vector quantization (VQ) [4] and dynamic time warping (DTW) [5] are representative examples of the earliest non-parametric techniques for text-independent and text-dependent speaker recognition, respectively. However, in recent years, classification methods for speaker recognition have centered around statistical approaches, with hidden Markov models (HMMs), Gaussian mixture models (GMMs) and support vector machines (SVMs) being representatives, and artificial neural networks (ANN) [6]. Generative models, such as VQ and GMM, estimate the feature distribution within each speaker, while discriminative models, such as SVM and ANN, focus on modeling the boundary between speakers [1].

The early standard statistical approaches rely on extracting low-dimensional input feature vectors. Spectral features, such as Mel-frequency cepstral coefficients (MFCC) are frequently used as they are easy to compute, capture detailed characteristics of individual speakers, and thus yield good performance [1,7]. Next, methods that incorporate i-vectors [8] and joint factor analysis (JFA) [9] showed promising results. Moreover, standard machine learning approaches, such as SVM and k-nearest neighbors (KNN), performed well in combination with MFCCs [10]. Still, it is observed that MFCC could fail when speech is introduced to real-world noises [11]. On the other hand, speech characteristics such as prosodic features, are less discriminative and easier to impersonate [12].

Even though standard statistical methods have led to significant progress in the SR field, most of these algorithms perform well only with test utterances in neutral speaking style, while they struggle to recognize speakers in more natural, emotional speech, which greatly limits their applications. For example, in research works presented in [10,13,14], when neutral speech is used in training and emotional speech for testing, the system fails significantly, with accuracy decreasing by more than 50% for some emotions. When the same emotional state is used for both training and testing, the classification scores reach better results [13], but recording emotional datasets could be demanding.

The speaker embedding representation, mainly i-vectors and x-vectors, are also used extensively for speaker recognition studies [15]. The advantage of these techniques lies in capturing speaker information robust to various external factors such as channel variation and background noise. Sarma et al. [16] proposed an approach to transform the i-vectors containing speaker-specific information into an emotion invariant space by creating emotion invariant speaker embedding. The proposed method led to an improvement in accuracy over the average speaker model-based framework with different emotions.

Incorporating deep neural networks (DNN) into systems based on hand-crafted features [1], as well as systems where DNNs are used to directly capture features, brought new possibilities into the area [11]. The latter approach introduced more flexibility in the overall SR process as it removes the necessity of preprocessing the raw audio data and thereby losing possibly valuable information. Lukic et al. [17] created a simple convolutional neural network to learn the features from raw spectrograms for the speaker identification task. They also introduced speaker clustering by using the activations of post-convolutional layers of pre-trained speaker identification CNNs to cluster the unknown speakers.

CNN-based models can be found in several other studies as a part of the architecture for speaker or emotion recognition. McLaren et al. built a SR system as a CNN that performed computation of senone probabilities and compared it with an i-vector approach [18]. Shafik et al. [19] proposed a CNN-based model for speech identification that works on the spectrograms of audio signals and their radon transforms. The model they propose provides high performance in terms of accuracy when the signal is exposed to noise, such as musical interference or the speech of another speaker. In [20], a lightweight CNN-based speech emotion recognition (SER) system using speech spectrograms was presented. With low computational complexity, the experimental results demonstrated that the system achieves a better recognition performance than the state-of-the-art SER systems. Anvarjon et al. [21] built a novel end-to-end CNN with a multi-attention module (MAM) for age and gender recognition from speech signals, with high classification accuracy results. These examples outline the importance of developing and enhancing deep learning models, especially CNN-based models for different speech processing tasks.

Most high-performable CNN architectures are complicated, deep networks, with intensive memory and processing requirements. As a consequence, a lot of these architectures are hard to integrate into real-time systems, especially when computation resources are highly limited, as in the case of smartphones [11]. Dai et al. [11] studied the performance of the VGGVox model on audio signals with background noises as well as signals recorded in controlled, noised-reduced environments. They introduced the model quantization process based on the affine mapping scheme and studied the applicability of the quantized CNN for real-life applications. While the computational requirements of the VGGVox model could be reduced without a serious performance cost, there is still space for the optimization of a CNN for usage in different environmental conditions. It can be concluded that there is a lack of research that analyzes the performance of constrained CNN models for speaker recognition tasks in general, including the case of aroused emotional state of a speaker.

In this paper, we propose a constrained CNN model for speaker recognition, which performs classification by processing speech spectrograms. The model we propose is designed following two ideas. Firstly, we wanted to design a robust model, which can operate in the case of an aroused emotional state in a speaker. Filters of rectangular kernel size recently became popular for emotion recognition tasks [20,22], so we decided to analyze and exploit them for the SR task. Kernels of such shape could better follow the shape of fast temporal changes of speech, embedded in spectrograms, which may lead to the reduction in the required network depth for high classification accuracy.

The idea of simplicity was further carefully considered as the emerging market of Internet of Things (IoT) devices is rapidly growing. Thus, we design constrained models and analyze their performance as such models can be implemented on small edge devices in IoT systems for real-time processing. Quantization appears as an effective tool for neural network reduction and there exist various approaches [23,24,25,26,27,28,29,30,31,32]. Here, we analyze post-training reduction in CNN weights using one of three popular quantization techniques: floating point eight (FP8) format, ternary quantization, and binary quantization. FP8 approach is chosen as it can provide performance almost equal as in the case of the full-precision network, whereas ternary and binary quantization provide the highest compression rates among scalar quantization techniques. Experiments are performed on the recently recorded Serbian emotional amateur corpus (SEAC), which is recorded by amateur speakers using mobile phones. The dataset consists of five predefined emotional styles: neutral, anger, fear, sadness, and joy.

The rest of the paper is organized as follows. In Section 2 we provide a description of the proposed architecture, including a full-precision convolutional neural network. Next, quantized CNN is described in detail in Section 3. In Section 4, we provide the experimental results, performance of the full-precision and quantized models as well as comparison with other models. Finally, the advantages and disadvantages of the proposed model are summarized in Section 5.

## 2. Proposed Speaker Recognition Architecture

With the rapid development and wider availability of powerful hardware in the previous decade, end-to-end neural networks became popular for speech signal processing. Commonly, spectrograms can be extracted from speech signals as a 2D visual representation of signal energy at different frequencies over time, and they can be further used for network training and classification [17,19,20,21,33,34]. For such extraction, we exploit short-term Fourier transform.

Before running the procedure for spectrogram image creation, input audio files are preprocessed. In the preprocessing stage, silence regions are detected using the algorithm described in [35] and removed from the input files. After silence removal, files are normalized to the maximum range.

**Figure 1:**
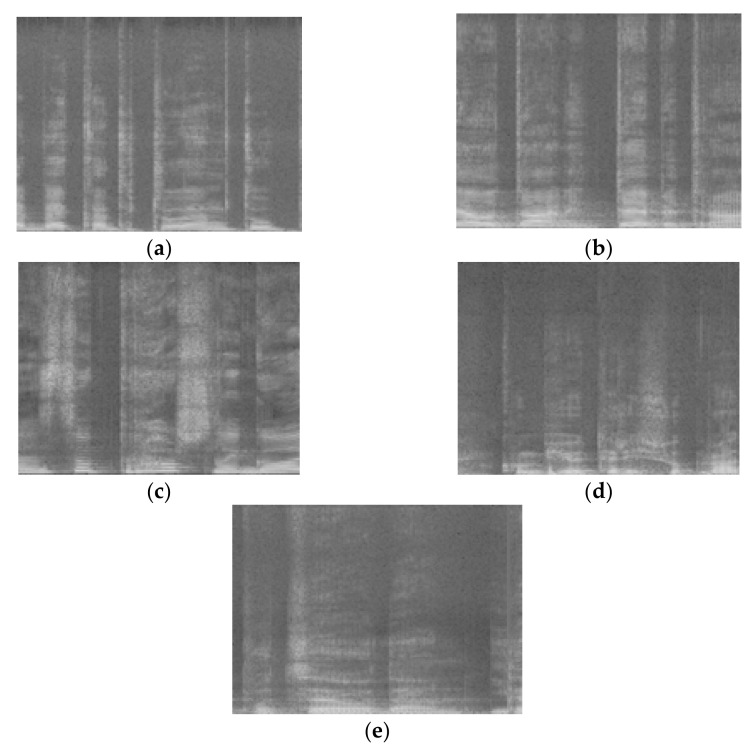
Examples of spectrograms for various styles of emotional speech obtained from the SEAC database: (**a**) neutral; (**b**) anger; (**c**) joy; (**d**) fear; and (**e**) sadness.

In the next step, files are divided into segments whose length is set to 1 s. If the last segment is shorter than 0.8 s, that segment is removed from the training set. If the last segment’s length is between 0.8 s and 1 s, it is extended to 1 s by replicating samples from the beginning of the segment. Each segment is then converted to the grayscale spectrogram image. Spectrograms are calculated using a 32 ms Hanning window with a time shift of 16 ms, so that there is an overlap among neighboring spectrograms of 50%. Finally, spectrogram images are saved in .png format resized to 128 × 170. Examples of created spectrograms can be seen in Figure 1.

The proposed full-precision convolutional neural network is described in Table 1.

Basically, the model consists of two convolutional layers followed by two max pooling layers: a fully-connected layer and the output layer. The shape of the input spectrograms is set to (128, 170, and 1) where the first two parameters correspond to the spatial resolution of an image, whereas the third indicates that the image is grayscale. The first convolutional layer has 16 kernels of size (9, 3) and it is followed by the rectified linear units (ReLu) activation function and a max pooling layer of size (2, 2). The second convolutional layer has 32 kernels of size (3, 1). Similarly to the first convolution layer, this layer is also followed by the ReLu activation function and a max pooling layer of size (2, 2). It can be observed that the kernels in the first convolutional layer have larger dimensions compared with the second convolutional layer and that the height of the kernels is larger than the width. The aim of the rectangular shape is more precise in capturing quick temporal changes, whereas kernel size reduction in the second layer should lead to better detection of discriminative features [22]. Next, the fully-connected layer has 128 nodes, whereas the output layer has 23 nodes, which is equal to the number of speakers used from the SEAC database. Finally, we use the softmax activation function after the output layer. Between the fully-connected layer and the output layer, we use dropout regularization with the parameter 0.2. The total number of parameters of the network is 4,994,039.

## 3. Quantized CNN

Deployment of neural network models to the embedded devices depends on several characteristics of the model, primarily on the achieved accuracy, processing time, and available memory. Most of the contemporary models are based on deep learning techniques that can provide high accuracy but at the expense of increasing memory and processing time. Thus, the quantization of neural network models is becoming an extremely important step from the standpoint of model deployment.

The design of quantizers is not uniquely determined and it strongly depends on the desired quality of reconstructed data as well as the complexity. Quantization refers to a process that maps values from the input set of data, which can be infinite in general, to the output set that consists of a fixed number of representative levels. Depending on the number of input samples that are processed at the same time, quantizers can be classified as vector and scalar. However, the vector quantizer’s design is more complex, so scalar quantization solutions are primarily analyzed for neural network compression tasks. In this section, we will describe three popular fixed scalar quantization approaches for quantizing neural networks. In the following sections, we will demonstrate the robustness of these approaches in the proposed CNN network, deployed for SR using emotional speech not seen in training.

### 3.1. The 8-Bit Floating Point Quantization

Floating point arithmetic is standardized within the IEEE 754 technical standard [26]. According to the standard, there are three basic binary formats, with encoding in lengths of 32 bits, 64 bits, and 128 bits. Commonly, 32 bits format is referred to as full-precision or single precision, whereas 64 bits format is a double precision and 128 bits is a quadruple precision format. Floating point 8 (FP8) arithmetic refers to a floating point format of a reduced precision [27,28], which exploits 8 bits for encoding an input sample. This format type is sometimes denoted as a minifloat format and represents a popular reduced precision alternative alongside a half-precision (16 bits) format. As minifloats have reduced precision, they are not well suited for general purpose arithmetic but they can be used for special purposes, such as to design constrained neural networks.

FP8 design can be carried out following the principles of the IEEE 754 standard. According to the standard, floating point format is specified by three components: sign, exponent, and mantissa. If we consider that there is one bit ‘s’ to encode a sign, *e* bits to encode exponent *E* ($a_{1}a_{2}\ldots a_{e}$) and *m* bits to encode mantissa *M* ($b_{1}b_{2}\ldots b_{m}$), a real number *x* can be represented in a floating point format as:

(1) $$x={\left(sa_{1}a_{2}\ldots a_{e}b_{1}b_{2}\ldots b_{m}\right)}_{2}$$

The exponent *E* and mantissa *M* can be calculated as:

(2) $$E={\left(a_{1}a_{2}\ldots a_{e}\right)}_{2}=\sum_{i=0}^{e-1}a_{e-i}2^{i}\;$$

(3) $$M={\left(b_{1}b_{2}\ldots b_{m}\right)}_{2}=\sum_{i=0}^{m-1}b_{m-i}2^{i}\;$$

Finally, the number *x* represented in the floating point format can be calculated as [32]:

(4) $$x={\left(-1\right)}^{s}2^{E_{b}}\left(1+\frac{M}{2^{m}}\right)\;$$

where *E_b_* = *E*—bias denotes the biased exponent. It should be noted that *M* can take values from 0 to $2^{m}-1$ whereas *E_b_* can take values from $-2^{e-1}$ to $2^{e-1}-1$. The value of bias and ranges of exponent and mantissa *M* depend on the number of bits reserved to represent exponent and mantissa.

In this paper, we discuss two FP8 formats. Firstly, we will examine the effects of applying a common (*s*, *e*, *m*) = (1, 4, 3) format, further denoted with FP8_v1. In this case, bias = 8, so that the biased exponent can take values from −8 to 7 whereas mantissa can take values from 0 to 2^3^–1 = 7. The second format that we analyze here is defined as (*s*, *e*, *m*) = (1, 5, 2) and it became popular recently as a part of a hybrid method for DNN training and inference [27]. In this case, bias = 16, the bias exponent takes values in the range from −16 to 15, whereas mantissa can take values from 0 to 2^2^–1 = 3. This format is further denoted as FP8_v2.

Let us consider an input sample *x*_1_. Determination of an FP8 representation for the given input sample can be done using Equation (4) and the following steps:

Step 1: Find the parameter s, following the rule:
  (5) $$s=\left\{\begin{array}{ll} 0 & x_{1}\geq 0\\ 1 & x_{1}<0 \end{array}\right.\;$$
Step 2: Find the biased exponent value *E_b_* by calculating the binary logarithm of the input sample:
  (6) $$E_{b}=\left\lfloor \log_{2}\left(\;\left|x_{1}\right|\;\right)\right\rfloor \;$$
  where $\left\lfloor \cdot \right\rfloor$ denotes the floor rounding function. If $E_{b}>2^{e-1}-1$, set $E_{b}=2^{e-1}-1$. Similarly, if $E_{b}<-2^{e-1}$, set $E_{b}=-2^{e-1}$.
Step 3: Find the mantissa *M* value as:
  (7) $$M=round\left(2^{m}\left(\frac{\left|x_{1}\right|}{E_{b}}-1\right)\right)\;$$
Step 4: Calculate the quantized value using Equation (4).

### 3.2. Binary Quantization

Fixed scalar binary quantization is the simplest scalar quantization model, which encodes an input real-valued sample *x* using 1 bit only. This way, maximal compression is achieved regarding scalar quantization techniques. Here, we exploit a symmetric binary quantizer, so that a binarized sample *x_b_* is obtained using the following rule [23]:

(8) $$x_{b}=\mathrm{Sign}(x)=\left\{\begin{array}{ll} +1 & x\geq 0\\ -1 & x<0 \end{array}\right.$$

Besides such deterministic binarization function, stochastic binarization function can be found in the literature, but such a model is harder to implement as it requires specific hardware [23].

### 3.3. Ternary Quantization

Ternary quantization was introduced to the neural network quantization task to reduce accuracy loss that occurs in binary quantization, by introducing a zero as an additional representative level. If we assume that *x* is present at the quantizer’s entrance, quantized value *x_t_* is obtained using the following rule [25]:

(9) $$x_{t}=\left\{\begin{array}{ll} y_{1} & x>\Delta_{1}\\ 0 & \Delta_{2}\leq x\leq \Delta_{1}\\ y_{2} & x<\Delta_{2} \end{array}\right.\;$$

where Δ_1_ and Δ_2_ represent decision-making thresholds. Here, we apply a symmetric ternary scalar quantizer, so that *y*_1_ = −*y*_2_ = *y*. Furthermore, we simplify design additionally by setting the absolute value of decision thresholds to a half value of the representative levels’ absolute value, so that the final design is:

(10) $$x_{t}=\left\{\begin{array}{ll} y & x>y/2\\ 0 & -y/2\leq x\leq y/2\\ -y & x<-y/2 \end{array}\right.$$

The optimal value of representative levels *y* strongly depends on the statistics of the neural network weights, i.e., statistics of the data that should be quantized. In this paper, we determine representative levels empirically, considering achieved classification accuracy and signal-to-quantization noise ratio value of reconstructed weights. This is relatively simple as we design a post-training quantization model and the total number of parameters is relatively small. Some advanced statistical models can be found in [25].

## 4. Results and Discussion

The evaluation of the proposed method is performed on the new Serbian emotional speech database, SEAC, which is soon to be made publicly available. This database is recorded by amateur speakers using mobile phones. Each subject could perform recording in up to five predefined emotional styles: neutral, anger, fear, sadness, and joy. Although there are recordings of 55 different speakers in the database, we used recordings from 23 (11 male and 12 female) speakers, who recorded speech in all five emotional styles. There are 60 sentences per recorded emotion. Commonly, sentences are 2–4 s long. All the recordings are resampled to 44.1 kHz and represented with 16 bits. As the goal is to design a robust model which can operate in various emotional styles and datasets consisting of several different emotional styles that are rare, we only used a neutral emotional style for training, whereas testing is done for each emotional state, separately. In order to minimize the bias inherent in the evaluation, we performed *k*-fold cross validation. Since we decided to use about 80% of the available spectrograms of neutral emotional style for training and 20% for validation, we performed five-fold cross validation. The number of spectrograms in each fold is not precisely equal, as we considered an equal number of recorded sentences per speaker in each fold, whereas the duration of recordings might vary. The precise number of spectrograms per fold is presented in Table 2.

To evaluate the performance of the proposed model, we consider average classification accuracy, F1 score, precision, and recall. It should be noted that the last three metrics are weighted. The results are shown in Table 3.

By observing the results from Table 3, it can be concluded that all five folds provide similar performance. The largest difference among folds is only 1.05% in terms of classification accuracy, and 0.02 in terms of weighted F1 score, precision, and recall. For further consideration, we choose the fifth fold, as its performance is closest to the average performance.

In Table 4, we summarize the number of spectrograms used for training and validation of the neutral emotional style (fifth fold) and testing of the other emotional styles. Although the same number of sentences was used for each emotion within the testing phase, the number of spectrograms per emotion is not the same, since different styles are also correlated also with different temporal changes, which is partly reduced by deleting silence from the recorded files in pre-processing, as is described in Section 2.

**Figure 2:**
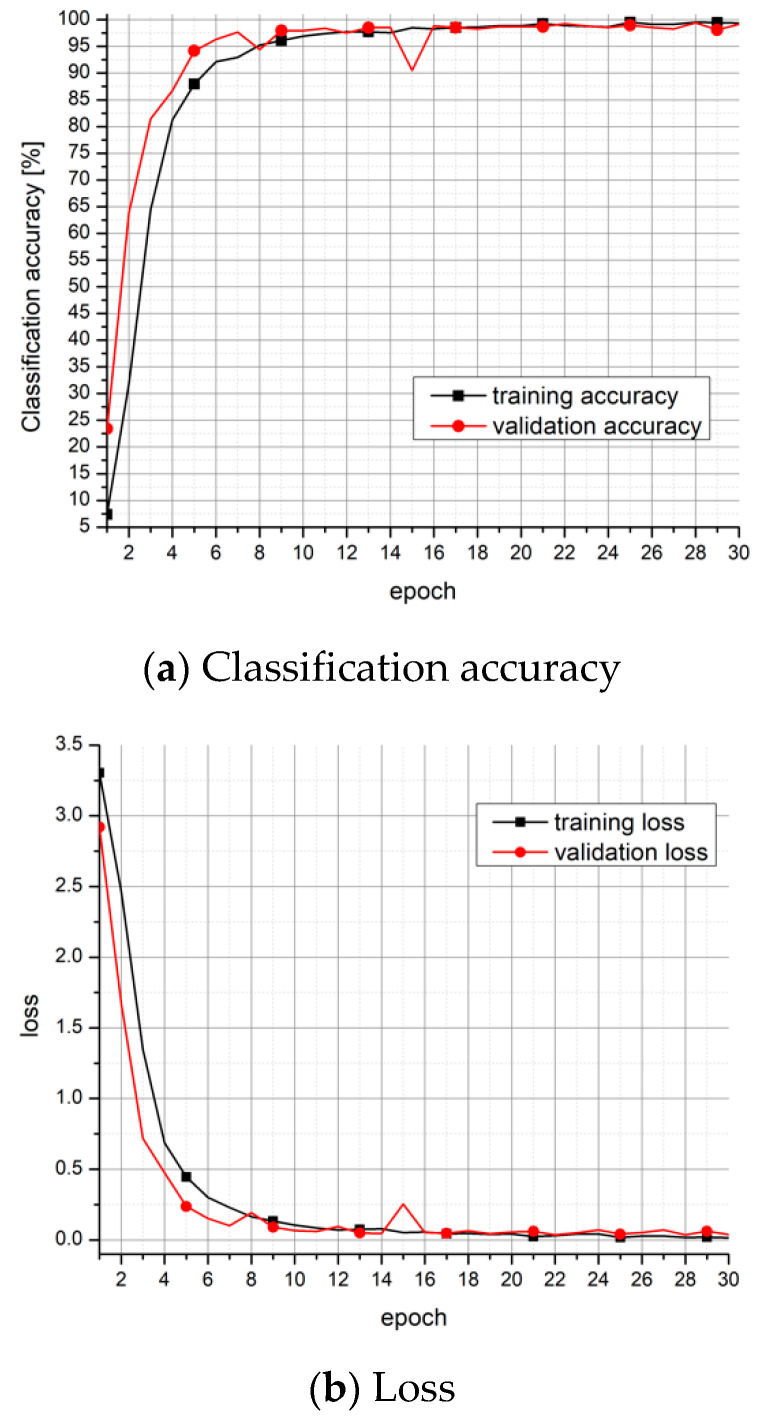
Training and validation performance of the full-precision CNN model.

It should be noted that there is no difference between the validation and testing sets for neutral style, as we performed *k*-folds cross validation and chose the fold whose performance is closest to the average performance. Figure 2 shows classification accuracy and loss for the first 30 epochs of training and validation on neutral speaking style.

Table 5 shows model predictions performance in the case of the trained full-precision model and after applying quantization methods described in Section 3. Predictions are made on all five types of recorded emotions: neutral, anger, fear, sadness, and joy. Furthermore, we present signal-to-quantization noise ratio (SQNR) between full-precision and quantized weights as the second performance measure, which provides a deeper understanding of applied quantization methods. SQNR is defined with:

(11) $$\mathrm{SQNR}=10\log_{10}\left(\frac{\sigma_{w}^{2}}{D_{w}}\right)=10\log_{10}\left(\frac{\frac{1}{N}\sum_{i=1}^{N}{\left(w_{i}-\mu \right)}^{2}}{\frac{1}{N}\sum_{i=1}^{N}{\left(w_{i}-w_{i}^{q}\right)}^{2}}\right)$$

where *D_w_* is weight distortion introduced during the quantization process, $w_{i}^{q}$ are quantized, $w_{i}$ are the original values of weights, *μ* is the mean value of original weights, whereas *N* is the total number of weights.

As it was already demonstrated, the proposed full-precision model achieves an accuracy of 99.12% on the validation dataset. The trained model is further used for the SR task in the case of emotional speech, and the results are shown in Table 5. It can be observed that there is a degradation of the performance in the case of emotional speech, as could be expected. However, achieved accuracy in the case of anger, fear, and joy is about 85%, which can be considered as a very good accuracy, taking into account that the model is not trained for such an environment. The largest degradation is noticed in the case of sadness. Such an observation can be explained by the fact that the speech rate of sadness is slower than in the other emotions [36], and that maybe even wider rectangular kernels should be used in this particular case.

By observing the performance of constrained models, one can notice that there is only negligible degradation of achieved accuracy in the case of both FP8 configurations compared with the full-precision model, whereas the required memory is reduced by almost four times. Considering minor differences among achieved classification accuracy results and SQNR values from Table 6, it could be seen that although the SQNR value for the FP8_v1 model with parameters (*s*, *e*, *m*) = (1, 4, 3) is higher compared with the FP8_v2 model with parameters (*s*, *e*, *m*) = (1, 5, 2), the achieved classification accuracy is the same for neutral, fear, and sadness, and slightly worse in the case of anger and joy, achieving the same level of compression. By further observing precision, recall, and the F1 score for these two FP8 models from Table 7, Table 8 and Table 9, the difference is not noticeable. Additional interesting observations can be noted for the results in the case of anger. It can be observed that both the FP8 quantized models provide slightly higher classification accuracy compared with the full-precision model in the case of anger, which could be considered as a contradiction as it is expected that after quantizing weights and introducing degradation, accuracy should be the same or reduced. Such unusual behavior can be explained by the fact that the model is trained only for neutral speech and that such a model is not optimal for other emotions. After applying FP8, we have introduced very small changes of weights, which coincided with hypothetical model learning in this case. The fact that these are small changes is reflected in high SQNR values for both FP8 configurations (Table 6). Similar observations can also be noticed in the case of ternary quantization and anger style, although higher distortion is introduced during the quantization process.

Ternary and binary quantization provide much higher compression ratios, reducing storage requirements of the full-precision model for almost 16 and 32 times, respectively. These compression-efficient techniques introduce the largest distortion, which can be seen from Table 6, that leads to the further degradation of classification accuracy. However, we underline that the average degradation of classification accuracy in the case of ternary quantization is only 1.51% and that the differences are lesser in the case of emotional speech not seen during the training phase, except sadness, highlighting the robustness of the proposed model. After considering a set of representative levels *y* = {1, 1/2, 1/4, 1/8, 1/16, 1/32}, we have chosen *y* = 1/16 as a representative level for ternary quantization since it provides the best accuracy and SQNR for our model.

In the case of binary quantization, the average degradation compared with the full-precision model on the set of five emotions is 5.544%, which is in accordance with the introduced degradation for other datasets [24].

Besides classification accuracy and SQNR, we observe weighted precision, recall, and F1 score as performance measures. These results are presented in Table 7, Table 8 and Table 9. Similarly, as in the case of classification accuracy, the full-precision model in the case of neutral speaking style provides excellent results and there is a degradation for the other emotions. The worst results can be noticed in the case of sadness for all three measures, which is in accordance with classification accuracy results. In the end, it can be observed that the performance of constrained models in the case of both FP8 versions is similar as in the case of the full-precision model, whereas a certain performance degradation exists in the case of ternary and binary quantization.

### Comparison with Other Models

The aim of this section is to provide comparisons of the proposed constrained CNN model with other state-of-the art CNN models and other machine learning techniques.

First of all, we analyzed the CNN model proposed in [17]. It can be seen that Lukic et al. proposed the model which includes twice the number of kernels in both convolutional layers, as well as an additional fully-connected layer. The summary of the model from [17], prepared for processing the SEAC dataset, is provided in Table 10.

By comparing the total number of parameters of the proposed model (Table 1) with the model from [17] (Table 10), it can be observed that the proposed model has 3.15 times fewer parameters so that it is much less complex and it requires much less memory.

In order to provide a detailed and fair comparison and to explore the robustness of rectangular kernels, we have also applied the proposed quantization methods to the model from [17]. The results are presented in Table 11 and Table 12.

**Figure 3:**
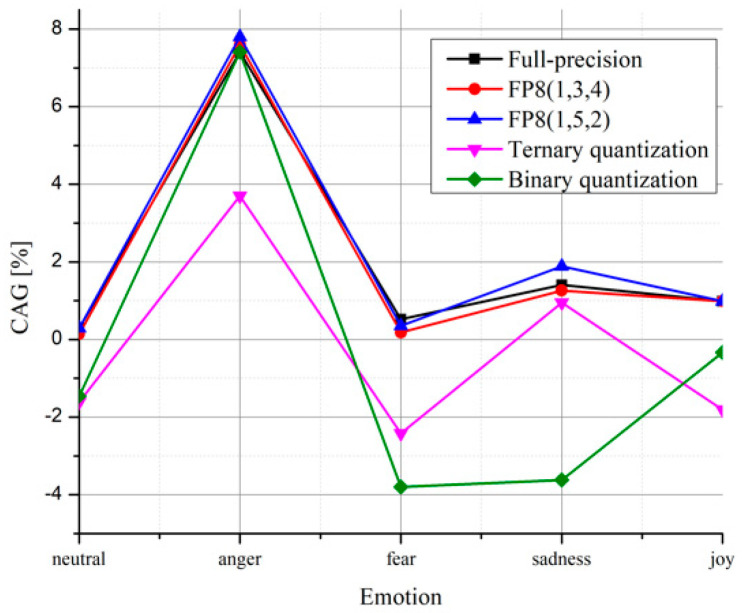
Classification accuracy gain over the model from [17].

By observing Table 11 and Table 12, similar observations could be made as in the case of the proposed model. In order to compare the achieved accuracy using the proposed model and the model from [17] and later with the models from [10,37], we define classification accuracy gain as an improvement of the proposed model over the compared model as:

(12) $$\mathrm{CAG}\;={\;\mathrm{ac}}_{p}-{\mathrm{ac}}_{c}$$

where ac_p_ represents the accuracy of the proposed model, whereas ac_c_ is the accuracy of the compared model. The positive values of CAG indicate better model performance of the proposed model. CAG values are given for all analyzed models and all emotional styles in Figure 3, providing a detailed comparison with the model from [17]. We can highlight that the proposed full-precision model provides better performance for all emotions, although the proposed network has far fewer parameters, demonstrating the suitability of rectangular kernels. By analyzing quantized CNN models, it can be seen that the proposed model would achieve better performance than the model from [17] for all emotional states in the case of both FP8 formats. However, better results in the case of ternary quantization are achieved only in the case of anger and sadness, whereas anger is the only emotional style for which better results are achieved in the case of binary quantization. Such behavior is not unusual, as the model from [17] has more than three times more parameters and the performance of low-precision models also rely on the model depth. However, more parameters and larger depth lead to longer processing and higher storage capability demands.

Although the model from [17] is a more complex CNN architecture than the proposed one, there exist far more complex networks in the literature. On the other hand, the aim of the paper is to propose a simple constrained solution, which could perform fast recognition and which could find application in IoT systems. Nevertheless, we also performed a comparison with a VGGish-based architecture, trained on the SEAC dataset. The VGGish-based model uses mel-spectrograms as input, but for the purposes of comparison we adjusted it to our usage scenario. The network is created following the descriptions from [37,38] and is summarized in Table 13.

It could be seen that the model consists of six convolutional layers, four max pooling layers, two fully connected layers, and the output layer. The total number of parameters is more than 205 million, which is about 41 times more demanding than the proposed model in terms of memory resources. The performance of the model is presented in Table 14.

By observing the results for the VGGish-based model and the proposed model, it could be seen that the proposed model achieves better classification accuracy for all emotions than the VGGish-based model, except fear. As the VGGish-based network has 41 times more parameters than the proposed model, quantization of such network is not worth analyzing for this research, as binary quantized VGGish-based architecture, obtained after reducing full-precision weights 32 times, would require more memory than the proposed full-precision model.

**Figure 4:**
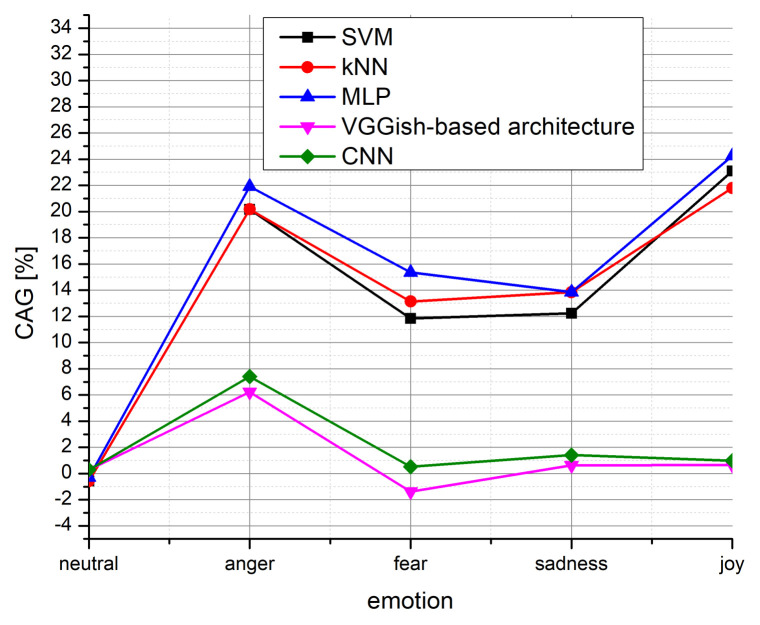
Classification accuracy gain over the various models: SVM, kNN and MLP [10]; VGGish-based architecture [37]; CNN model from [17].

Besides comparing the proposed model with other CNN models, let us observe the achieved performance of SVM, KNN, and multilayer perceptron (MLP) on the same dataset. These results are already presented in [10]. By observing Table 3 from [10], it can be seen that these methods provide great classification accuracy in the case of neutral speech, even slightly better than the model we propose. However, when deploying such models trained to the neutral speech on other emotional styles, there exists a huge degradation, much larger than in the case of the proposed CNN model. The achieved classification accuracy gain of the proposed full-precision model compared with the SVM, KNN, MLP, and full-precision CNN model from [17] and VGGish-based architecture from [37] is presented in Figure 4.

## 5. Summary and Conclusions

In this paper, we proposed constrained convolutional neural network models for speaker recognition tasks. The models were designed following two ideas. Firstly, we wanted to design a robust model, which can operate in the case of an aroused emotional state of speakers. For such a task, we exploited filters of a rectangular kernel shape unlike common CNN models for speaker recognition, which exploit kernels of a square shape. Secondly, the idea of simplicity was considered carefully in order to design a model that can be implemented on small edge devices in IoT systems for real-time processing. The proposed architecture considers spectrograms of a recorded speech, so that a short 1-s-long utterance is enough for detection. Furthermore, we made constrained models by analyzing several techniques for neural network weights’ quantization.

The experiments were performed on the closed set of speakers provided in the SEAC database, which consists of five different emotional styles, recorded in the Serbian language. We demonstrated that the proposed unconstrained CNN model provides high classification accuracy, averaging 99.248% in the case of neutral speech and about 85% in the case of all other observed emotions except sadness, which is detected with 79.84% accuracy. Furthermore, we analyzed the system performance in the case of constrained representation of weights, using 1-bit, 2-bit, and 8-bit quantization. The achieved results suggest that the proposed constrained models provide results very close to the one obtained using the full-precision 32-bit model; there is only negligible difference in the case of 8-bit representation, whereas the difference in the case of the ternary quantization model is up to 3.3%, while degradation in the case of the binary quantization model is up to 10.55%. Unlike the full-precision model and FP8 models, the performance of 1-bit and 2-bit quantized models is more dependent on the model depth. For both ternary and binary quantization, the worst results are achieved in the case of sadness, which turned out to be the emotional style with the worst degradation.

In the end, we have compared the performance of the proposed model with the performance of other state-of-the-art CNN-based speaker recognizers, which have about three times more and 41 times more network parameters. We have concluded that these two full-precision models provide similar or worse performance compared with the proposed full-precision model, excepting the case of fear and the network from [37]. To highlight the importance of the proposed solution, we also demonstrated the gain of the proposed model over the SVM, KNN, and MLP solutions. As the proposed model performs classification based on a single input spectrogram, i.e., on a 1-s-long utterance, we intend to analyze compromising solutions in the future. It can be expected that the implementation of majority voting techniques should increase classification accuracy, at the expense of increasing complexity and processing time.

## Figures and Tables

**Table 1:** The proposed CNN model.

Layer	Arguments	Number of Parameters
Convolution2D	Filters = 16, kernel size = (9, 3), input shape (128, 170, 1)	448
MaxPooling2D	Pool size = (2, 2)	
Convolution2D	Filters = 32, kernel size = (3, 1)	1568
MaxPooling2D	Pool size = (2, 2)	
Flatten		
Dense_1	Nodes = 128	4,989,056
Dropout	Rate = 0.2	
Dense_2	Nodes = 23	2967
Total number of parameters		4,994,039

**Table 2:** The number of spectrograms in folds.

Fold	Number of Spectrograms
1	607
2	587
3	593
4	583
5	684
Total	3054

**Table 3:** Cross-validation performance of the proposed full-precision model.

Fold	Classification Accuracy (%)	Weighted F1	Weighted Precision	Weighted Recall
1	99.51	1.00	1.00	1.00
2	99.49	1.00	0.99	0.99
3	99.66	1.00	1.00	1.00
4	98.46	0.98	0.98	0.98
5	99.12	0.99	0.99	0.99
Average	99.248	0.994	0.992	0.992

**Table 4:** The number of spectrograms used per emotion within the experiment.

	Emotion	Number of Spectrograms
Training	Neutral	2370
Testing	Neutral	684
	Anger	513
	Joy	608
	Fear	579
	Sadness	635

**Table 5:** Classification accuracy of the proposed full-precision and quantized CNN models.

	Classification Accuracy (%)				
	Emotion				
Proposed Model	Neutral	Anger	Fear	Sadness	Joy
Full-precision	99.12	86.16	84.46	79.84	85.69
FP8 (1, 4, 3)	99.12	86.35	84.46	79.84	85.69
FP8 (1, 5, 2)	99.12	86.55	84.46	79.84	85.86
Ternary quant.	97.22	86.35	83.07	76.54	84.54
Binary quant.	94.01	83.82	77.37	69.29	83.06

**Table 6:** SQNR for various quantization models.

	SQNR (dB)
	Proposed Model
FP8 (1, 4, 3)	30.98
FP8 (1, 5, 2)	25.58
Ternary quant. (1/16)	5.90
Binary quant.	−31.483

**Table 7:** Weighted precision of the proposed full-precision and quantized CNN models.

	Weighted Precision				
	Emotion				
Proposed Model	Neutral	Anger	Fear	Sadness	Joy
Full-precision	0.99	0.88	0.88	0.84	0.89
FP8 (1, 4, 3)	0.99	0.88	0.88	0.84	0.89
FP8 (1, 5, 2)	0.99	0.88	0.88	0.84	0.89
Ternary quant.	0.97	0.88	0.87	0.82	0.87
Binary quant.	0.95	0.86	0.84	0.72	0.87

**Table 8:** Weighted recall of the proposed full-precision and quantized CNN models.

	Weighted Recall				
	Emotion				
Proposed Model	Neutral	Anger	Fear	Sadness	Joy
Full-precision	0.99	0.86	0.84	0.80	0.86
FP8 (1, 4, 3)	0.99	0.86	0.84	0.80	0.86
FP8 (1, 5, 2)	0.99	0.86	0.84	0.80	0.86
Ternary quant.	0.97	0.86	0.83	0.77	0.85
Binary quant.	0.94	0.84	0.77	0.69	0.83

**Table 9:** Weighted F1 score of the proposed full-precision and quantized CNN models.

	Weighted F1 Score				
	Emotion				
Proposed Model	Neutral	Anger	Fear	Sadness	Joy
Full-precision	0.99	0.86	0.85	0.79	0.86
FP8 (1, 4, 3)	0.99	0.86	0.85	0.79	0.86
FP8 (1, 5, 2)	0.99	0.86	0.85	0.79	0.86
Ternary quant.	0.97	0.86	0.83	0.75	0.85
Binary quant.	0.94	0.84	0.77	0.67	0.83

**Table 10:** The CNN model from [17].

Layer	Arguments	Number of Parameters
Convolution2D	Filters = 32, kernel size = (4, 4), input shape (128, 170, 1)	544
MaxPooling2D	Pool size = (4, 4), strides = (2, 2)	
Convolution2D	Filters = 64, kernel size = (4, 4)	32,832
MaxPooling2D	Pool size = (4, 4), strides = (2, 2)	
Flatten		
Dense_1	Nodes = 230	15,662,310
Dropout	Rate = 0.5	
Dense_2	Nodes = 115	26,565
Dense_3	Nodes = 23	2668
Total number of parameters		15,724,919

**Table 11:** Classification accuracy of the CNN model from [17]: full-precision and additionally quantized scenarios.

	Classification Accuracy (%)				
	Emotion				
CNN Model from [17]	Neutral	Anger	Fear	Sadness	Joy
Full-precision	98.83	78.75	83.94	78.43	84.70
FP8 (1, 4, 3)	98.98	78.75	84.28	78.58	84.70
FP8 (1, 5, 2)	98.83	78.75	84.11	77.95	84.87
Ternary quant.	98.83	82.65	85.49	75.59	86.35
Binary quant.	95.47	76.41	81.17	72.91	83.39

**Table 12:** SQNR for various quantization models applied to the model from [17].

	SQNR (dB)
	CNN from [17]
FP8 (1, 4, 3)	30.94
FP8 (1, 5, 2)	25.54
Ternary quant. (1/16)	5.219
Binary quant.	−33.94

**Table 13:** The VGGish-based architecture.

Layer	Arguments	Number of Parameters
Convolution2D	Filters = 64, kernel size = (3, 3), strides = (1, 1), input shape (128, 170, 1)	640
MaxPooling2D	Pool size = (2, 2), strides = (2, 2)	
Convolution2D	Filters = 128, kernel size = (3, 3), strides = (1, 1)	73,856
MaxPooling2D	Pool size = (2, 2), strides = (2, 2)	
Convolution2D	Filters = 256, kernel size = (3, 3), strides = (1, 1)	295,168
Convolution2D	Filters = 256, kernel size = (3, 3), strides = (1, 1)	590,080
MaxPooling2D	Pool size = (2, 2), strides = (2, 2)	
Convolution2D	Filters = 512, kernel size = (3, 3), strides = (1, 1)	1,180,160
Convolution2D	Filters = 512, kernel size = (3, 3), strides = (1, 1)	2,359,808
MaxPooling2D	Pool size = (2, 2), strides = (2, 2)	
Flatten		
Dense_1	Nodes = 4096	184,553,472
Dense_2	Nodes = 4096	16,781,312
Dense_3	Nodes = 23	94,231
Total number of parameters		205,928,727

**Table 14:** Classification accuracy of the full-precision VGGish-based architecture.

	Classification Accuracy (%)				
	Emotion				
VGGish-Based Architecture	Neutral	Anger	Fear	Sadness	Joy
Full-precision	98.83	79.93	85.84	79.21	85.03

## Data Availability

Not applicable.
